# Skin cancers in albinos in a teaching Hospital in eastern Nigeria - presentation and challenges of care

**DOI:** 10.1186/1477-7819-8-73

**Published:** 2010-08-25

**Authors:** Kingsley O Opara, Bernard C Jiburum

**Affiliations:** 1 Plastic Surgery Division, Department of Surgery, Imo State University Teaching Hospital, Orlu, Imo State, Nigeria

## Abstract

**Background:**

Albinism is a genetic disorder characterized by lack of skin pigmentation. It has a worldwide distribution but is commoner in areas close to the equator like Nigeria. Skin cancers are a major risk associated with albinism and are thought to be a major cause of death in African albinos. Challenges faced in the care of these patients need to be highlighted in order to develop a holistic management approach with a significant public health impact. The aim of the study was to determine the pattern of skin cancers seen in Albinos, and to highlight problems encountered in their management.

**Method:**

Case records of albinos managed in Imo state University teaching Hospital from June 2007 to May 2009 were reviewed. The data obtained was analyzed using descriptive statistics.

**Results and discussion:**

In the period under review, albinos accounted for 67% of patients managed for primary skin cancers. There were twenty patients with thirty eight (38) lesions. Sixty one percent of the patients were below 40 years. Average duration of symptoms at presentation was 26 months. The commonest reason for late presentation was the lack of funds. Squamous cell carcinoma was the commonest histologic variant. Most patients were unable to complete treatment due to lack of funds.

**Conclusion:**

Albinism appears to be the most important risk factor in the development of skin cancers in our environment. Late presentation and poor rate of completion of treatment due to poverty are major challenges.

## Introduction

Albinism is a genetic disorder characterized by lack of skin pigmentation. Its mode of inheritance is thought to vary, depending on the type. The oculocutaneous type is considered autosomal recessive, and the ocular variant sex linked [1].

Albinism has a worldwide distribution, but is said to be commoner in regions of the world closer to the equator, with greater penetration of the sun's ultraviolet radiation [2]. It has an estimated frequency of 1 in 20000 in most populations with the highest incidence of 6.3 per 1000 reported among the Cuna Indians [2,3]. In Africa, incidences ranging from 1 in 2,700 to 1 in 10,000 have been reported in various studies [4-7].

Melanin is a photo protective pigment, protecting the skin from the harmful effects of ultraviolet radiation. Its deficiency therefore predisposes to various degrees of actinic injury to the skin. These include sunburns, blisters, Centro facial lentiginosis, ephelides, solar elastosis, solar keratosis, basal cell carcinomas and squamous cell carcinomas [5,8]. Squamous cell carcinoma has been reported to be the commonest skin malignancy seen in albinos [9,10]. In Africa the incidence of squamous cell carcinoma in the general population ranges from 7.8 to 16% of all diagnosed skin malignancies [4]. In the African albino, the risk of developing these malignancies in comparison to the general population has been reported to be as high as 1 to 1000 [11,12]. In Aquaron's 15 year review of albinos in Cameroon [13], he reported solar induced squamous cell carcinoma as being the commonest cause of death in albinos.

In this article, we are reviewing the albinos managed for skin cancers in our center over a two year period, with emphasis on the pattern of presentation and management problems.

## Background

Imo State University Teaching Hospital is located in Orlu, a sub-urban town in Eastern Nigeria. It is one of the few tertiary health institutions offering Plastic surgery services to the Eastern and Southern parts of Nigeria.

Nigeria is the most populous nation in sub-Saharan Africa and the most populous black nation in the world with a population of about 140 million people. It lies in the peri-equitorial region, between latitudes 4°and 14° north of the equator with a high degree of sunshine all through the year. Thus her population like all those living around the equator is exposed to a high degree of ultraviolet radiation all year round.

## Patients and Method

Hospital records of patients with Albinism managed for skin cancers at the Imo State University Teaching Hospital from June 2007 to May 2009 were reviewed. Data on age, sex, occupation, duration of symptoms, distribution of lesions, treatment offered and rate of completion of treatment were extracted. Data were analyzed using descriptive statistics.

## Results

A total of twenty (20) albinos with thirty eight (38) lesions were managed in the period under review, giving an average of 1.9 lesions per patient. These accounted for 67% of all primary skin cancers managed in our center in the period under review.

**Figure 1:**
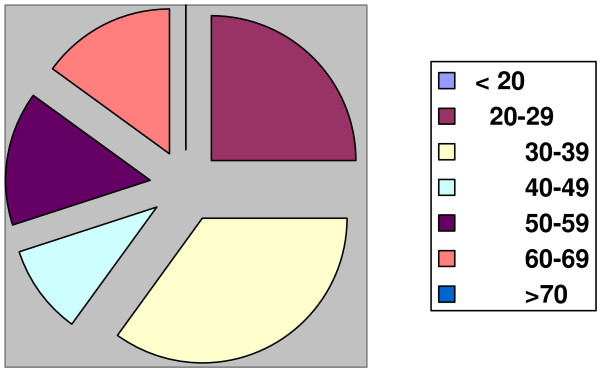
**Age Distribution**.

**Figure 2:**
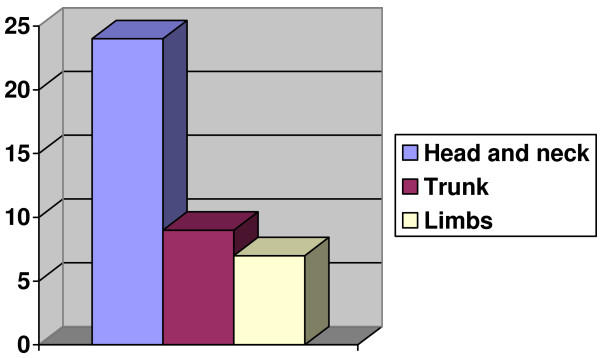
**Distribution of lesions**.

There were 10 males and 10 females giving a Male to Female Ratio of 1:1 (Table 1). Their ages ranged from 21 years to 67 years with twelve (61%) of the patients below the age of 40 years (Figure 1). Most of the patients presented late, with an average time at presentation of 26 months. Fifteen (75%) of the patients were outdoor workers involved in semi-skilled and unskilled labour. The commonest part of the body involved was the head and neck, while the limbs were least affected (Table 1, Figure 2). The commonest histologic variant was Squamous cell Carcinoma; 32 lesions. 5 were basal cell carcinomas and one baso-squamous.

**Table 1:** Patient data

Patient No.	Age in yrs/sex	Duration of symptoms	Site	Size	Treatment
1	55/M	13 mth	Post. Trunk	7 × 5 cm	EXC.+Flap
		6 mth	Neck	4 × 5 cm	EXC. + DC
		4 mth	Neck	3 × 2.5 cm	EXC. + DC
2	42/M	34 mth	Post. Trunk	14 × 16 cm	Rad
3	39/M	24 mth	Post. Trunk	16 × 12 cm	EXC.+Flap
		11 mth	Post Trunk	2 × 3 cm	EXC. + DC
4	37/F	4 mth	Forearm	9 × 7 cm	EXC. + SSG
		3 mth	Fore head	1 × 1.5 cm	EXC. + DC
5	67/F	20 mth	Post. Trunk	6 × 4.5 cm	EXC.+Flap
		14 mth	Ant. Trunk	5 × 4 cm	EXC. + DC
		4 mth	Fore arm	3 × 4 cm	EXC. + DC
6	33/M	38 mths	Nose/cheeks/ eyelids	14 × 12 cm	Rad
7	21 M	9 mth	Upper lip	4 × 4.5 cm	EXC.+Flap
8	58/F	18 mth	Upper lip	8 × 6 cm	EXC.+Flap+Rad
9	52/M	13 mth	Nose	2 × 3 cm	EXC.+Flap
10	22/M	48 mth	Nose	4 × 4 cm	EXC.+Flap+Rad
11	37/F	11 mth	Nose	4 × 3.5 cm	EXC.+Flap+Rad
					
		4 mth	Cheek	2 × 1.5 cm	EXC. + DC
12	21/F	5 mth	Nose	2 × 2 cm	EXC. + Flap
		4 mth	Forehead	2 × 1.5 cm	EXC. + DC
13	28/F	42 mth	Cheek	14 × 11 cm	Rad
		13 mth	Ant. Trunk	6 × 4.5 cm	Rad
14	63/F	36 mth	Cheek	12.5 × 9 cm	Defaulted
		8 mth	Cheek	2 × 1.5 cm	Defaulted
		8 mth	Fore head	2 × 2.5 cm	Defaulted
15	46/F	40 mth	Cheek	10 × 8 cm	EXC.+Flap+Rad
		7 mth	Fore head	2 × 2 cm	EXC. + DC
16	30/M	22 mth	Fore head	9 × 6 cm	EXC. + Flap+ SSG + Rad
		8 mth	Ear	2 × 2.5 cm	EXC. + Flap
		7 mth	Cheek	3 × 1.5 cm	EXC. + DC
17	37/F	10 mth	Forearm	5 × 4 cm	EXC. + Flap
18	38/M	26 mth	Upper arm	18 × 12 cm	Defaulted
		16 mth	Post. Trunk	6 × 8 cm	Defaulted
19	28/F	3 mth	Fore head	5 × 4 cm	EXC.+Flap
		1.5 mth	Fore head	1 × 1.5 cm	Exc. + DC
		6 mth	Ant. trunk	5 × 3 cm	EXC,+ DC
20	67/M	168 mth	Fore arm	16 × 8 cm	EXC.+SSG+Rad
		162 mth	Fore arm	16 × 8 cm	EXC.+SSG+Rad
					

KEY: EXC: Excision, DC: Direct Closure, Rad: Radiotherapy, SSG: Split thickness Skin Graft.

Excision of tumour with a margin and primary reconstruction was our commonest modality of treatment (29 lesions). This was usually combined with adjuvant radiotherapy for recurrent lesions as well as deep seated lesions. Fourteen (70%) of the patients did not complete their treatment or were lost to follow up shortly after commencement of treatment. Seven (50%) of these were patients requiring adjuvant radiotherapy. Most had complained of lack of funds at the time of referral for radiotherapy.

## Discussion

Albinos accounted for 67% of patients presenting with cutaneous malignancies in our centre, making it the single most important risk factor in the development of skin cancers in our environment.

Non melanotic skin cancers are generally commoner in the middle aged and elderly. In albinos however these cancers are known to present earlier [14,15]. In his review of 1000 Nigerian albinos, Okoro AN [5] found none above the age of 20 to be free of solar induced premalignant or malignant skin lesions. A similar finding was also reported by J Launde et al [16] in their review of 350 albinos in Dar-es-Salam. In that study, the peak age of patients with advanced skin cancers (greater than 4 cm in diameter) was the 4^th ^decade of life. In this study, 61% of our patients were in the 3^rd ^and 4^th ^decades of life.

Skin cancers are indeed a major cause of morbidity amongst albinos in the tropics. These patients from a young age face a raging battle against these cancers; a battle the African albino often appears to lose [13]. These cancers have been reported to be the major cause of death amongst African albinos. Okoro AN[5] found only 6.3% of 1000 albinos reviewed, above the age of thirty years while the study in Dar-es-Salam [16] found less than 10% of their study population above 30 yrs of age; figures consistently lower than the expected figures in the general population.

From available reports, skin cancers in albinos are preventable [2,5]. There is therefore a need for early institution of skin protective measures in these patients. To achieve this, public enlightenment and education are essential. The albino needs to avoid undue exposure to the sun, use sunscreens and wear protective clothing (avoid sleeveless attires and use long sleeved attires as much as possible) during periods of sun exposure. The wearing of bowler hats, which in this environment have been produced from cheap and available raffia, is quite effective. Government and private employers of labour should engage their albino staff in indoor rather than outdoor duties.

Fifteen (75%) of our patients were either engaged in peasant farming, outdoor trade or a type of menial job with increased risk of solar exposure. This is similar to the findings by J Launde et al [16] in Dar-es salsm, where only 12% had indoor occupations. Okoro AN [5] succinctly captures the interaction between clinical and social factors in heightening the solar exposure risks of the albino: He says "Myopia and other ocular defects retard the progress of many albinos in school and they eventually drop out to seek disastrous menial outdoor occupations" These apart from heightening the sun exposure risks of the patients, are often poor paying jobs. These patients therefore lack the financial capability to handle their health needs. It is therefore needful for health insurance schemes to provide cover for the informal sector to which most of these patients belong.

Late presentation was a prominent feature in this study. The average duration of symptoms at presentation was 26 months. Poverty and ignorance were the main reasons for this. Some however presented early to a healthcare facility, but were offered inadequate or ineffective forms of treatment, only to be referred late. There is therefore a need for persons with albinism as well as healthcare providers at all levels of care to be enlightened on the health needs of the albino.

The head and neck region was the commonest site of these cancers followed by the trunk, and then the limbs. This has been the pattern reported in other studies [9,10,15,17] and is similar to the pattern of non-melanotic skin cancers seen in non albinos of Caucasian descent. As in the Caucasians, sun exposure is thought to be the major aetiologic factor for cutaneous cancers in African albinos [9,10,18] and may be responsible for this pattern of distribution. However unlike in whites where basal cell carcinoma is by far the commonest histologic variant, [19,20] in albinos, as was seen in this study, the squamous cell variety appears to be commoner [9,10,15]

**Figure 3:**
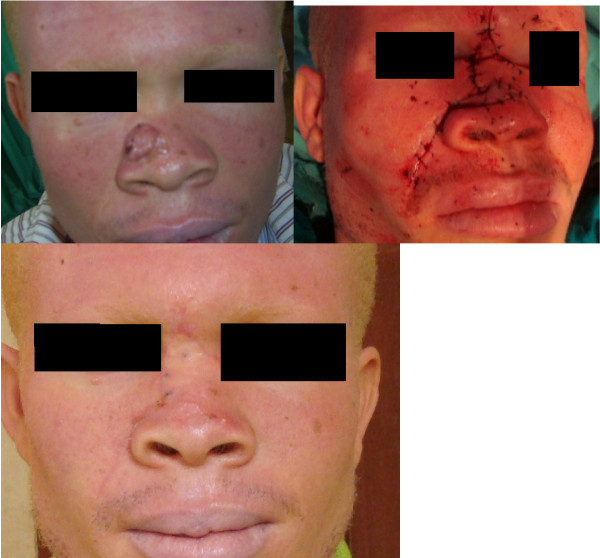
**Patient No. 10: Multiple flap reconstruction of the nose following tumour resection**.

**Figure 4:**
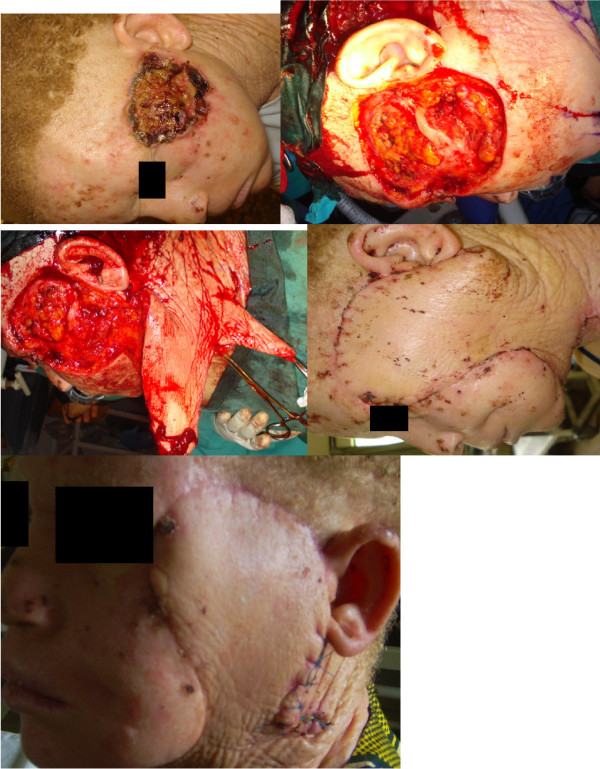
**Patient No. 15: Multistaged tumour excision with cheek reconstruction**.

**Figure 5:**
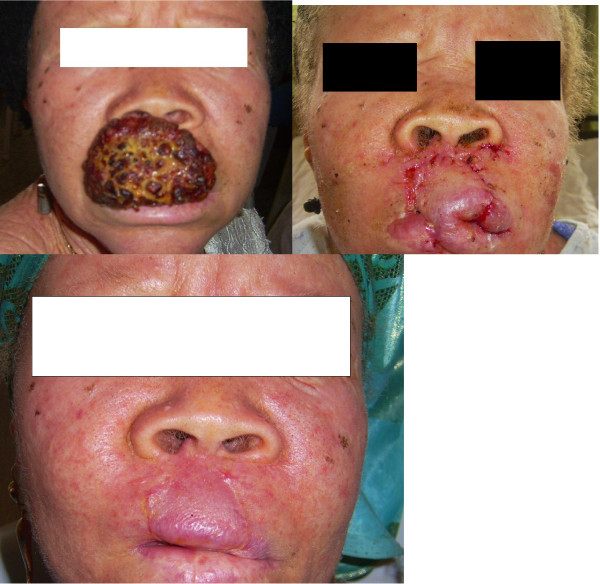
**Patient No. 8: Multistaged tumour excision with lip reconstruction using bilateral cheek advancement with a central abbe flap**.

**Figure 6:**
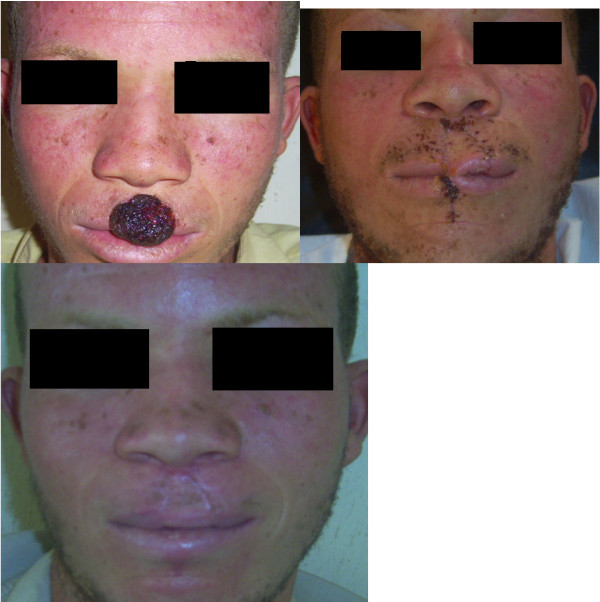
**Patient No. 7: Multistaged tumour excision with lip reconstruction using bilateral cheek advancement with a central abbe flap**.

**Figure 7:**
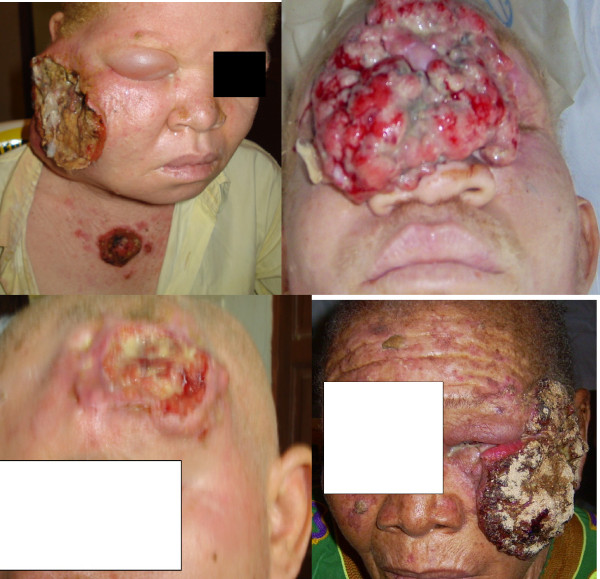
**Patient No. 13, 6, 16, 14 in serial order**.

With these patients presenting late and majority of the lesions affecting the head and neck, defects following resection were usually complex and affected multiple aesthetic units and or major proportions of single aesthetic units. Reconstruction was therefore often complex and multi-staged (Figures 3, 4, 5, 6 and 7). This on a background of poverty and scarcity of treatment funds posed a further challenge to patient care as a significant number of patients were unable to complete treatment due to lack of funds.

## Conclusion

Squamous cell carcinoma is the commonest non-melanotic skin cancer seen in albinos in our environment. Most patients are young adults and early institution of sun protective measures is key to prevention.

Late presentation is a problem. To address this, the albino as well as the health care providers at all levels of care need to be enlightened on the cancer risks of the albino. A centralized registry for albinos with free annual skin checks would improve early detection and treatment, hence reducing the morbidity and mortality of skin cancers in these patients

There is a need for the government as part of its social obligation to provide treatment funds for these mainly poor patients. Advocacy groups apart from providing the much needed public enlightenment may also assist in seeking for treatment subsidies/grants for the albino patient.

## Consent

Written informed consent was obtained from patients for publication of images with a promise to conceal their identity. A copy of the written consent is available for review by the editor-in-chief.

## Competing interests

The authors declare that they have no competing interests.

## Authors' contributions

KOO conceived the study, participated in the design and coordination of the study and drafted the manuscript. BCJ participated in designing the study and drafting the manuscript. All authors read and approved the final manuscript.
